# Antibacterial silver nanoparticle decorated gallium metal-organic frameworks for odontogenic infections

**DOI:** 10.1038/s41598-026-47319-7

**Published:** 2026-04-04

**Authors:** Fellype Diorgennes Cordeiro Gomes, Diptomit Biswas, Logan C. Eisaman, Mary Cristina Ferreira Alves, Severino Alves Júnior, Scott H. Medina

**Affiliations:** 1 Department of Fundamental Chemistry, Federal University of Pernambuco, Cidade Universitária, Recife, 50670 Brazil; 2 Department of Biomedical Engineering, Pennsylvania State University, University Park, PA 16802 USA; 3 Department of Chemistry, Paraíba State University, Campina Grande, Paraíba 58429 Brazil; 4 Huck Institutes of the Life Sciences, Pennsylvania State University, University Park, PA USA

**Keywords:** Oral infection, Gallium, Silver nanoparticles, Metal-organic frameworks, Biotechnology, Chemistry, Materials science, Microbiology, Nanoscience and technology

## Abstract

Surgical debridement of infected oral tissues can compromise maxillofacial aesthetics and anatomical function, thereby reinforcing the use of antibiotics as a first-line therapy. However, the escalation of multidrug resistance has accelerated the search for non-antibiotic antimicrobial platforms. In this study, bactericidal hybrid gallium–silver metal-organic frameworks (MOFs) were synthesized by nucleating the formation of silver nanoparticles at the surface of gallium MOF scaffolds. Spectroscopy studies confirmed the elemental composition of silver-gallium MOF hybrids, while X-ray diffraction showed that the crystalline framework was retained after silver incorporation. Thermogravimetric analysis further indicated that silver incorporation increased thermal decomposition temperatures of the complex, and reduced total mass loss from ~ 57% to ~ 47%, indicating anchorage of silver nanoparticles at the gallium MOF surface stabilizes the carrier nanostructure. Antibacterial assays demonstrated gallium-silver MOFs significantly inhibit the growth of the model oral pathogens *S. mitis* and *S. pneumoniae* at a dose of ≥ 3.9 µg/mL, a concentration found to be tolerated by human cellular models, through contact-dependent membrane disruption. Therapeutic formulations were additionally effective in clearing microbial biofilms grown on tooth enamel-mimetic hydroxyapatite discs. These proof-of-concept findings suggest that gallium–silver hybrid MOFs hold promise as an attractive topical antimicrobial with potential to rapidly clear odontogenic infections.

**Supplementary Information:**

The online version contains supplementary material available at 10.1038/s41598-026-47319-7.

## Introduction

Odontogenic infections result from outgrowth of bacterial pathobionts at the tooth surface and invasion into gingival tissues and adjacent bone structures^1^. When not adequately treated, localized oral lesions can quickly evolve into more severe pulpitis and periodontitis^1,2^. Standard therapeutic approaches typically involve administration of broad-spectrum antibiotics, along with surgical debridement for advanced infections. Although effective, these interventions carry significant limitations, including aesthetic concerns, functional impairments following surgery, and the emergence of antibiotic-resistant pathogens. These challenges underscore the need for fast-acting, non-antibiotic therapies that can be locally administered, potentially reducing, or even eliminating, the need for broad spectrum antibiotics and surgical intervention^3,4^.

Towards this goal, antimicrobial and antioxidant nanoparticles represent a promising alternative to conventional pharmaceutics in dental disease^5,6^. Metallic nanoparticles, including silver, gold, copper and titanium based formulations, have attracted particular attention due to their intrinsic antimicrobial activity, anti-biofilm effects and photocatalytic properties^7–10^. Among the array of available metals to choose from, gallium (Ga), a post-transition metal with iron-like properties,^11–13^ has been comparatively understudied, despite its potential to interfere with iron metabolism to inhibit bacterial growth. The complementary osteoinducing effects of Ga^3+^ ions suggest this metal represents an ideal therapeutic scaffold to clear dental infections and promote bone regrowth^12^. In particular, Ga has been reported to promote osteoblast proliferation via TRPM7/Akt signaling and differentiation through induction of alkaline phosphatase activity and calcium nodule formation, while also inhibiting osteoclasts^14,15^.

**Fig. 1:**
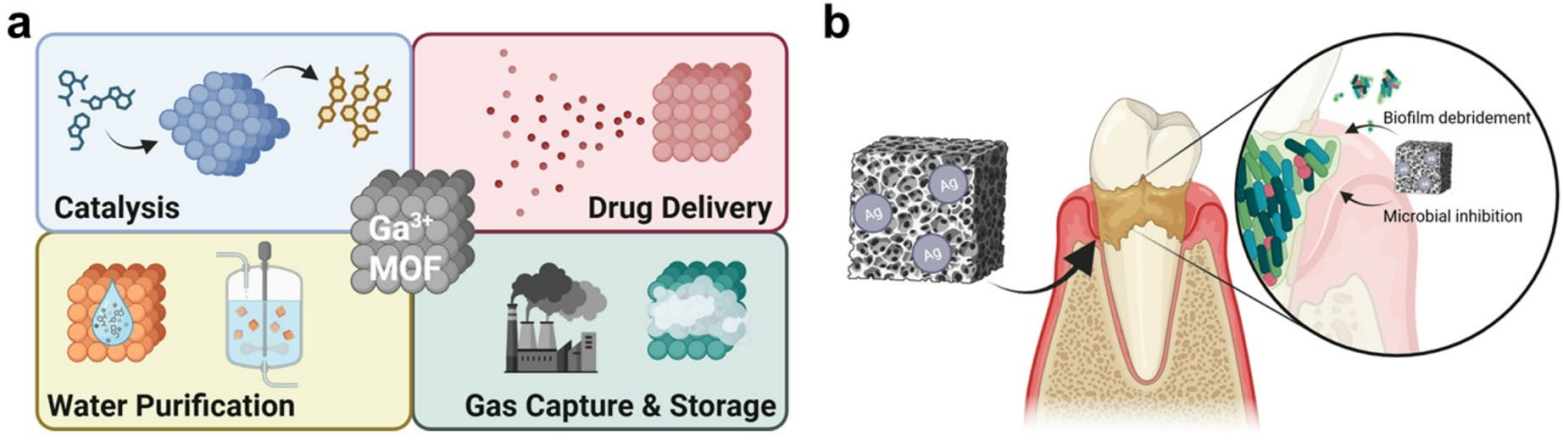
(**a**) Summary of the recent biomedical applications of Ga-MOFs. (**b**) Ga-MOFs functionalized with silver nanoparticles hold rapidly clear dental infections through microbial and biofilm inhibition, while promoting osteogenesis in the compromised oral tissues. Graphic created with BioRender.com.

However, Ga salts rapidly disassociate in biologic tissues, which limits the solubility and bioavailability of the free Ga³⁺ ions. To overcome this, Ga has been organized with various hydrophilic organic ligands to generate highly stable metal-organic frameworks (MOFs)^13,16–18^. These structures exhibit a wide range of advantageous properties, including thermal and mechanical stability, tunable porous structures for compound storage, and aqueous solubility^17,19–22^. Such features, combined with the diversity of available organic linkers, have expanded the use of Ga‑MOFs across biocatalysis, water purification, drug delivery, and gas separation (Fig. 1a). In antimicrobial applications, Ga‑MOFs not only disrupt iron‑dependent metabolic pathways but also promote intracellular reactive oxygen species (ROS) generation, which damages proteins, DNA, and lipids^18^. Additionally, their highly ordered architectures can act as light‑absorbing scaffolds that enhance photodynamic therapeutic efficacy^23^.

Although Ga‑MOFs exhibit intrinsic antibacterial properties, their activity alone is often insufficient to eradicate persistent infections, necessitating combination strategies. For instance, Liu et al. demonstrated that incorporating the antimicrobial peptide melittin into Ga‑MOFs significantly enhanced their efficacy against *S. aureus* compared to either agent alone in a skin wound infection model^24^. Building on this concept, we integrate the iron metabolism-disrupting activity of Ga-MOFs with antibacterial silver nanoparticles (AgNPs) to create hybrid frameworks with potent antimicrobial activity against the oral pathogens *Streptococcus mitis* and *Streptococcus pneumoniae* (Fig. 1b). AgNPs were selected as a complementary therapeutic component due to their multi-modal antibacterial mechanisms, including membrane disruption, DNA and protein damage, and ROS generation, which may synergistically enhance gallium’s bioactivity^25^. Additionally, AgNPs exert anti-biofilm activity, a critical feature for effective management of periodontal disease^26–28^. Yet, despite their significant therapeutic potential, AgNPs are generally water insoluble and therefore require delivery vehicles to enable effective oral application. Through structural and thermodynamic characterization studies, we show that AgNPs can be stably anchored to Ga-MOF scaffolds, producing water dispersible hybrids suitable for topical use. Functional assays confirm that these Ag-modified Ga-MOFs exhibit superior antibacterial performance compared to either component alone, while maintaining clinically relevant biocompatibility with human bone cells and gingival fibroblasts. These findings support the continued development of Ag-modified Ga-MOFs as an attractive therapeutic platform for rapid clearance of dental and maxillofacial infections without inducing significant further osteopenia.

## Materials and methods

### Materials

Gallium nitrate (Ga(NO_3_)_3_·9H_2_O), Polyvinylpyrrolidone (PVP), Silver nitrate (AgNO_3_), Mellitic acid (C_12_H_6_O_12_), Yeast extract, and MTT (3-(4,5-dimethylthiazol-2-yl)-2,5-diphenyl tetrazolium bromide) were purchased from Sigma-Aldrich. Sodium citrate (C_6_H_5_Na_3_O_7_·2H_2_O) was purchased from Sigma-Aldrich. Dimethyl sulfoxide (DMSO) was purchased from Fisher BioReagents, and Tryptic Soy broth purchased from MP Biomedicals. All reagents were of analytical grade and were used without any further purification.

### Synthesis of silver nanoparticles (AgNPs)

The synthesis of silver nanoparticles was conducted using the Turkevich–Lee–Meisel method^29^. An aqueous AgNO₃ solution (125 mL, 0.001 M) was heated to boiling, followed by dropwise addition (1 drop s⁻¹) of 1 mL sodium citrate solution (0.038 M) under vigorous stirring. The citrate-to-silver molar ratio (~ 0.30) was selected to promote rapid nucleation and effective electrostatic stabilization, limiting particle growth and aggregation and producing a particle size ~ 50 nm. The solution was maintained at boiling until a pale yellow color appeared, indicating nanoparticle formation. After reaction completion, the suspension was cooled gradually under stirring, washed repeatedly with distilled water, centrifuged, and dried at 50 °C.

### Synthesis of Ga-MIL-116

The Ga-MIL-116 metal–organic framework (MOF) was synthesized by a hydrothermal method according to the procedure described by Volkringer et al.^30^. In brief, in a Teflon-lined stainless steel autoclave, 5 mL of distilled water, 400 mg of gallium nitrate (Ga(NO₃)₃·9 H₂O), and 200 mg of mellitic acid were added. The reactor was hermetically sealed and heated at 210 °C for 24 h. After the reaction period, the autoclave was allowed to cool naturally to room temperature (25 °C). The resulting solid was collected, washed several times with methanol and distilled water to remove unreacted species, and then centrifuged and dried at room temperature.

### Synthesis of silver-modified Ga-MIL-116 (Ga-MIL-116@AgNPs)

The synthesis of silver nanoparticles (AgNPs) was adapted from previously reported methods for metallic nanoparticles^29,31^. Initially, 125 mL of an aqueous silver nitrate (AgNO₃) solution (0.001 M) was heated under magnetic stirring until boiling (95–100 °C). At this temperature, a previously prepared aqueous suspension of Ga-MIL-116 (10 mg of Ga-MIL-116 dispersed in 1 mL of water) was added to the boiling solution and maintained under stirring for 5 min. Subsequently, 1 mL of a sodium citrate (Na₃C₆H₅O₇) solution (0.038 M) was added dropwise (approximately one drop per second) using a Pasteur pipette, acting as a reducing agent. Then, 10 drops of a polyvinylpyrrolidone (PVP) solution (0.05 g of PVP dissolved in 5 mL of water) were added to stabilize the nanoparticles. The gradual color change from pale yellow to amber indicated the formation of silver nanoparticles within the MOF structure. Finally, the material was collected and dried in an oven at 50 °C.

### Biophysical characterization

The crystalline structure of Ga-MIL-116@AgNPs was analyzed using powder X-ray diffraction (PXRD). Measurements were performed on a SmartLab diffractometer (Rigaku, Japan) with Cu Kα radiation (λ = 1.5406 Å). The diffraction patterns were collected over a 2θ range of 5–80°, with a scanning step size of 0.01°/min. UV–Vis absorption spectra were recorded on a Shimadzu UV-2600 spectrophotometer equipped with a diode array detector and integrating sphere. Measurements were conducted in the 200–800 nm wavelength range, with a spectral resolution of 1 nm, at 25 °C. Thermal behavior of Ga-MIL-116, Ga-MIL-116@AgNPs and AgNPs was evaluated using a Shimadzu DTG-60 H thermobalance or a Discovery TGA 550 (TA instruments). Samples were placed in either platinum or alumina pans and heated from room temperature to 900 °C at a rate of 10 °C/min under a nitrogen flow of 100 mL/min. FTIR spectra were acquired using a PerkinElmer Spectrum 400 spectrometer (Serial No. 82287) in the range of 4000–400 cm⁻¹. Samples were analyzed in the solid state using ATR mode. Particle size distribution and zeta potential were obtained using a NanoBrook Omni instrument. TEM imaging was performed to evaluate the morphology and spatial distribution of silver nanoparticles within the MOF matrix. Samples were prepared via drop-casting onto lacey carbon-coated copper grids and analyzed using a FEI Talos transmission electron microscope operating at 200 kV. For SEM samples, bacteria were grown and treated with MOF samples as previously described. The samples were passed through a 0.2 micron pore filter disc to trap bacteria, followed by fixation with glutaraldehyde (2.5% v/v) in phosphate buffer for 30 min followed by step-wise dehydration with 10, 25, 50, 75, 85, 95, and 2 × 100% solutions of ethanol. The discs were then dehydrated with a Leica EM CPD300 Critical Point Dryer (Leica) prior to being mounted on titanium stubs and sputter coated with a 4.5 nm layer of iridium prior to viewing. XPS characterization of Ga-MIL-116 and Ga-MIL-116@AgNP samples was performed using a Physical Electronics VersaProbe III instrument equipped with a monochromatic Al kα x-ray source (hν = 1,486.6 eV) and a concentric hemispherical analyzer. Charge neutralization was performed using both low energy electrons (< 5 eV) and argon ions. The binding energy axis was calibrated using sputter cleaned Cu (Cu 2p3/2 = 932.62 eV, Cu 3p3/2 = 75.1 eV) and Au foils (Au 4f7/2 = 83.96 eV)^32^. Measurements were made at a takeoff angle of 45° with respect to the sample surface plane. This resulted in a typical sampling depth of 3–6 nm (95% of the signal originated from this depth or shallower). Quantification was done using instrumental relative sensitivity factors (RSFs) that account for the x-ray cross section and inelastic mean free path of the electrons. The analysis size was ~ 200 μm in diameter. Peaks were charge referenced to CHx band in the Carbon 1s spectra at 284.8 eV for all samples.

### Antimicrobial testing

The sensitivity of bacterial strains against Ga-MIL-116, Ga-MIL-116@AgNPs, and AgNPs were evaluated against *Streptococcus mitis* (ATCC 49456) and *Streptococcus pneumoniae* (unencapsulated type 2 strain D39, obtained from the National Collection of Type Cultures, NCTC). Both bacterial strains were cultured in Trypticase Soy Broth (TSB) supplemented with 0.5% yeast extract at 37 °C under 5% CO₂. Stock suspensions of the materials were prepared at a concentration of 1000 µg/mL in 2% DMSO in modified TSB media and syringe filtered to sterilize before use. For the bacterial sensitivity assays, 100 µL of the stock suspension was placed in the first well of a 96-well microplate, followed by serial two-fold dilutions across the plate, each containing 50 µL of fresh modified TSB media. From each well, 50 µL was transferred to the next one, mixed, and 50 µL was discarded after the last dilution, resulting in a final volume of 50 µL per well. After the serial dilutions were completed, 50 µL of bacterial inoculum (OD_600_ = 0.02) were added to each well, reaching a total volume of 100 µL per well. Positive and negative controls consisted of 20% and 1% final DMSO volume ratio, respectively. Additionally, background controls for each dilution were added to monitor aggregation. The microplates were incubated at 37 °C. Bacterial growth was monitored by measuring the optical density at 600 nm (OD₆₀₀) using a Synergy H1 Biotek microplate reader at specific time intervals. Values were normalized relative to background control and plotted using GraphPad Prism. All assays were performed in triplicate and replicated three times (*n* = 9).

To evaluate potential changes in particle efficacy in an artificial saliva environment, Ga-MIL-116@AgNPs were pre-treated for various time points in artificial saliva (composition detailed below), followed by a single wash in sterile water before being redissolved in 500 µL of modified TSB media. Following protocols described above, antimicrobial susceptibility (MIC) was determined against *S. mitis*. Following bacterial addition, plates were incubated at 37 °C under 5% CO_2_ and OD_600_ was measured every 10 min for up to 15 h. Relative MIC was determined with respect to water pre-treatment for 2 h (*n* = 4).

Quantification of live and dead cell fractions was performed using the Invitrogen™ LIVE/DEAD BacLight Kit following manufacturer’s protocol. Log-phase bacterial cultures were diluted to an OD_600_ of 0.02 in sterile TSB media supplemented with 0.5% yeast extract. Stock suspensions of Ga-MIL-116 and Ga-MIL-116@AgNPs were prepared at a concentration of 125 µg/mL in modified TSB media, after dissolving them initially in 2% v/v DMSO. 250 µL of prepared bacterial suspensions were mixed with different volumes of either Ga-MIL-116 or Ga-MIL-116@AgNPs stock suspensions to achieve final treatment concentrations of 3.9, 15.6 and 62.5 µg/mL in 1.5 mL microcentrifuge tubes. Samples were incubated at 37 °C under a 5% CO_2_ atmosphere for 5, 15, 25 and 45 h, with independent tubes utilized for each time interval to ensure discrete sampling. Following incubation, bacteria was pelleted by centrifugation at 5000 g for 15 min and washed twice with 0.85% w/v sterile saline. 100 µL of the resuspended bacterial suspension were transferred to black 96-well microplates. A 2X staining solution in saline was prepared using the SYTO9 and PI fluorescent dyes from the kit, and 100 µL of this solution was added to each well. Following a 15-minute incubation at room temperature in the dark, green and red fluorescence were measured using a Synergy H1 Biotek microplate reader with excitation/emission wavelengths of 485/560 nm and 485/630 nm, respectively. Untreated bacterial suspensions and MOF suspensions in blank media (containing 1% v/v DMSO) were included as negative and background controls. Green/red fluorescence ratios were calculated and plotted in GraphPad Prism to denote live to dead bacterial fraction for all samples. The assay was performed twice independently, each with three technical replicates (*n* = 6).

Biofilm assays were performed by placing sterile hydroxyapatite (HA) discs (0.5-inch diameter) into 24-well plates along with 1 mL of modified TSB media, either as sterile controls or inoculated with bacteria to a final OD_600_ of 0.05. To promote biofilm formation, the media was supplemented with 0.25% w/v glucose and 1% w/v sucrose. Plates were then incubated at 37 °C under 5% CO_2_ for 12 h before treatment with Ga-MIL-116 or Ga-MIL-116@AgNPs (62.5 µg/mL final concentration) or blank supplemented TSB media for both sterile wells (background controls) and wells with bacterial biofilms. Following a 24-hour incubation at 37 °C under 5% CO_2_, media was carefully removed from all wells and 1 mL PBS were added to the sides of the well to wash biofilms. After removing PBS, HA discs were air-dried for 20 min on Kimwipes™ and then placed in new wells. 0.5 mL of 0.1% crystal violet (CV) solution in distilled water was added to the wells, and it was incubated for 20 min at 37 °C in the dark. Excess CV solution was removed, and discs were carefully washed with distilled water twice. 1 mL of 30% acetic acid in distilled water was added to all wells and plates were placed on a shaking incubator at 37 °C in the dark for 10 min. 100 µL aliquots were transferred from each well into a black 96-well microplate. Absorbance at 575 nm was measured using Synergy H1 Biotek microplate reader and normalized relative to background controls for both untreated and treated samples. Values were then plotted using GraphPad Prism and statistical significance was evaluated using one-way ANOVA (*p* < 0.05). A total of 5 technical replicates were utilized for this experiment.

### Silver release in artificial saliva

Artificial saliva was freshly prepared, as described in Gal et al.,^33^ by dissolving the following salts in 100 mL of distilled water in the given order: NaCl (12.56 mg), KCl (96.39 mg), urea (20 mg), Na_2_SO_4_ (33.65 mg), NaHCO_3_ (63.8 mg), NH_4_Cl (17.8 mg), CaCl_2_.2H_2_O (22.78 mg), KSCN (18.92 mg) and KH_2_PO_4_ (65.45 mg). The solution was sterilized using a 0.22 μm filter and its pH was adjusted to either 5.5 or 7 using 0.5 M HCl solution. To prepare artificial saliva with salivary enzymes, α-amylase from Bacillus sp. (Sigma-Aldrich^®^) was dissolved at a concentration of 20 U/mL. Ga-MIL-116@AgNPs stocks (1000 µg/mL) were prepared in sterile distilled water and diluted 1:4 in one of three artificial saliva solutions and added to a 24 well plate. Samples were incubated at 37 °C under orbital shaking (100 rpm) to mimic saliva flow for 2, 6 and 24 h. Ga-MIL-116@AgNPs stocks diluted 1:4 in water and blank water diluted 1:4 in artificial saliva solutions were used as negative and background controls, respectively.

To evaluate silver ion release from Ga-MIL-116@AgNPs, samples were placed in 1.8 mL microcentrifuge tubes, dissolved in artificial saliva, and at defined incubation times pelleted via centrifugation at 5000xg for 10 minutes. The release profile of Ag^+^ ions was determined using the chromogenic substrate 3,3’,5,5’-Tetramethylbenzidine (TMB). Briefly, 50 µL of supernatant was treated with 100 µL of 100 mM H_2_O_2_ in 0.1 M acetate buffer (pH 4) and 10 µL of 10 mM TMB stock in DMSO in a black 96-well microplate. Plates were incubated at 37 °C in the dark for 1 h before absorbance measurements at 652 nm using a microplate reader. Values were normalized relative to background controls and plotted on GraphPad Prism. 62.5 µg of free AgNPs dissolved in 50 µL of distilled water, treated similarly with H_2_O_2_ and TMB dye for 1 h, served as a positive control (*n* = 6).

### Reactive oxygen assay

Early log-phase bacterial cultures were diluted to an OD_600_ of 0.1 in sterile TSB media supplemented with 0.5% yeast extract. 50 µL of this suspension was added to the wells of a 96 well microplate in triplicate containing 1 µL of 1 mM DCFH-DA stock in DMSO, before addition of 50 µL of Ga-MIL-116 or Ga-MIL-116@AgNPs suspensions to achieve final concentrations of 1.95, 3.9, 7.81 and 15.63 µg/mL. Cells treated with blank media and 10 mM H_2_O_2_ served as negative and positive controls, respectively. Fluorescence measurements were recorded using a Synergy H1 Biotek microplate reader with an excitation/emission wavelength of 485/525 nm.

### Mammalian cell viability

Host cytotoxicity of Ga-MIL-116, Ga-MIL-116@AgNPs and AgNPs were determined in the human osteosarcoma MG-63 (originating from bone tissue - Rio de Janeiro Cell Bank, Rio de Janeiro, Brazil; passage 23) and gingival fibroblast cell lines (HGF1 CRL-2014™ from ATCC^®^). Both cell lines were cultured in Dulbecco’s modified Eagle’s medium (DMEM), supplemented with 10% fetal bovine serum (FBS), L-glutamine (2 mM) and penicillin/streptomycin (1%), and maintained in an incubator at 37 °C under 5% CO_2_. For MG-63, cell suspensions were seeded in 96-well plates at a density of 3 × 10^3^ and incubated overnight, while for HGF1, 5 × 10^3^ cells/well were seeded and incubated for 72 h, before being treated with different sample concentrations (3.0; 6.0; 12.0; 25.0; 50.0–100 µg/mL) for 24 h. Cells treated with blank media and 20% DMSO were used as negative and positive controls, respectively. After incubation with the treatment, 10 µL of MTT (3-(4,5-dimethylthiazol-2-yl)-2,5-diphenyltetrazolium bromide) diluted in DMEM (5 mg/mL) was added to each well and incubated for 3 hours in an incubator at 37 °C and 5% CO2. After removing the supernatant, 100 µL DMSO was added to each well and the plate was incubated in a shaking incubator for 10 min to dissolve the formazan crystals. Cell viability was measured via absorbance at 570 nm using a microplate reader. Cell viability was calculated as (A_treatment_ – A_positive_ control)/ (A_negative_ control – A_positive_ control) × 100%. These values were then plotted and fitted using nonlinear regression analysis in GraphPad Prism to calculate 50% cell inhibition concentrations (IC_50_). All experiments were performed at an *n* ≥ 3.

## Results and discussion

**Fig. 2:**
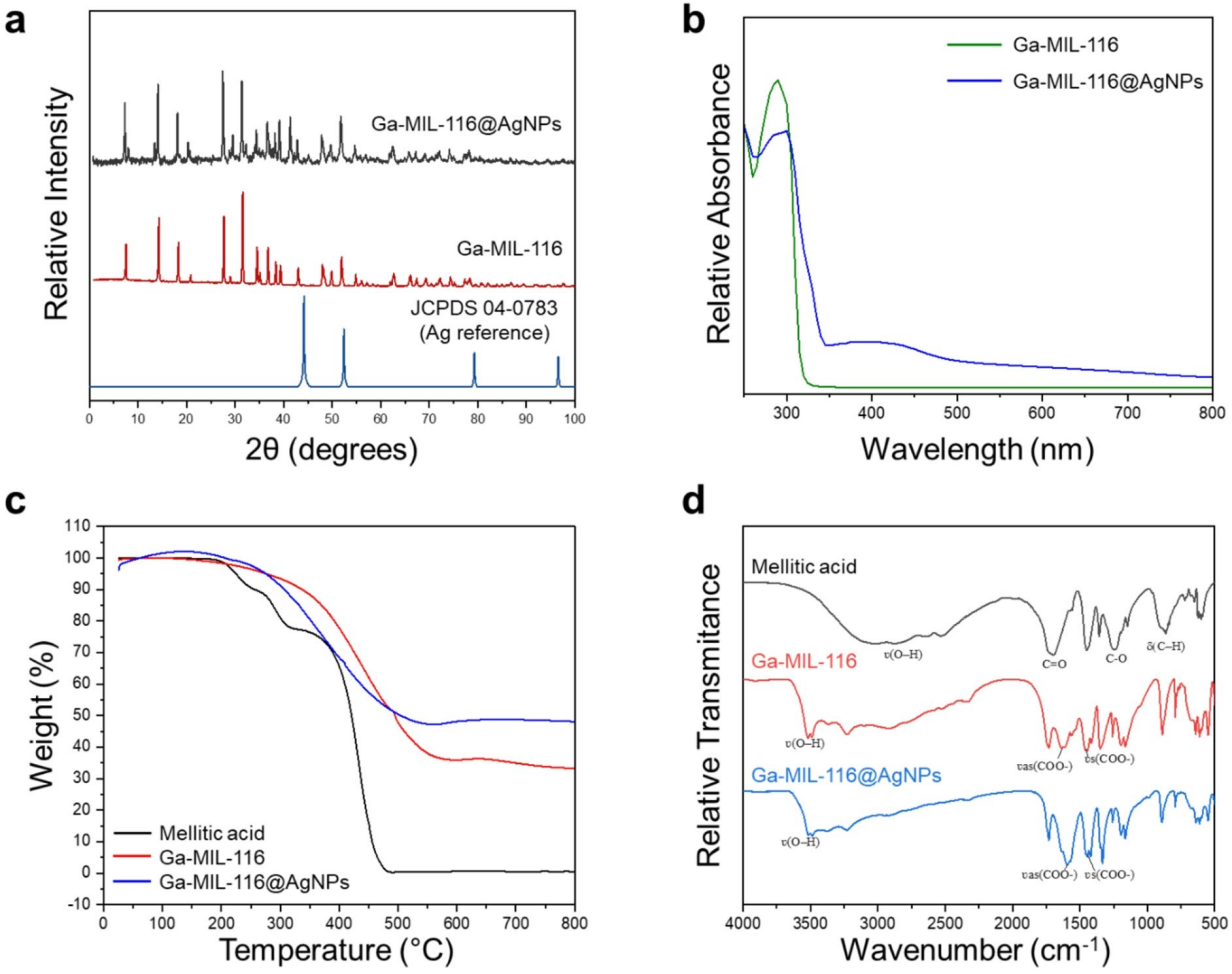
(**a**) Diffractograms of Ga-MIL-116 and Ga-MIL-116@AgNPs, relative to the silver nanoparticle reference (JCPDS 04-0783). (**b**) UV-Vis spectra of Ga-MIL-116 and Ga-MIL-116@AgNPs. (**c**, **d**) Thermogravimetric curves (**c**) and infrared absorption spectra (**d**) of Ga-MIL-116 and Ga-MIL-116@AgNPs. The organic linker mellitic acid is included as a reference control.

X-ray diffraction (XRD) analysis of the Ga MOF composite (Ga-MIL-116, Fig. 2a) confirms a highly crystalline structure consistent with an orthorhombic three-dimensional framework, in agreement with the crystallographic data reported for CCDC 929,715 and Volkringer et al.^30^ The diffractogram of Ga-MIL-116@AgNPs shows a similar overall diffraction pattern, indicating that the MOF structure is largely preserved after the incorporation of silver nanoparticles. However, a slight shift of the main peaks toward lower 2θ values, as well as peak broadening relative to pristine Ga-MIL-116, suggests subtle structural distortions in the crystalline framework caused by the presence of AgNPs. The shoulder at ~ 38°, small feature at 44°, and broad peak at 77°, in the Ga-MIL-116@AgNPs trace are consistent with the (111), (200), and (311) reflections of face-centered cubic silver (see control spectra in Supplementary Fig. 1, and reference JCPDS 04-0783 in Fig. 1a), confirming successful integration of the antibacterial metal species in the hybrid MOF.

To better assess the structural changes of the gallium framework, the unit cell parameters and volumes were calculated based on the experimental diffraction data using the crystallographic dataset CCDC-929,715 as a reference. The main diffraction peaks at 2θ = 10.26°, 15.43°, 18.49°, 20.37°, 25.46°, 26.49°, and 28.37°, corresponding respectively to the (002), (200), (202), (004), (204), (020), and (022) planes, were used in these calculations. The results indicate that both Ga-MOF materials adopt an orthorhombic configuration within the Cmcm space group (Supplementary Table S1). A reduction in the lattice parameters and corresponding unit cell volumes was observed compared to the theoretical values, with the contraction being less pronounced for Ga-MIL-116@AgNPs than for the pristine Ga-MIL-116. This behavior is consistent with the deposition of silver nanoparticles on the porous MOF surface, as previously reported^34^. We additionally performed UV/Vis spectrophotometry to interpret the optical properties of the structures and confirm the presence of bound silver ions (Fig. 2b). Spectra collected for the bare Ga-MIL-116 MOF showed a characteristic peak at 295 nm, corresponding to the π-π* transitions of the organic mellitic acid linker. This feature was diminished following AgNP complexation, and a broad peak spanning 350–500 nm emerged, suggesting the presence of silver bound to the MOF scaffold. This was further confirmed via X-ray Photoelectron Spectroscopy (XPS, Supplementary Figure S2), which showed that 12.7% of the total Ga-MIL-116@AgNP atomic surface composition is hybridized silver.

Next, thermogravimetric analysis (TGA) was performed to investigate the temperature-dependent mass variation of the samples to infer stability properties and water content. Figure 2c shows the mellitic acid ligand, as a control, begins to lose its adsorbed water molecules at ~ 150–200 °C, before undergoing a multistep thermal decarboxylation between 200 and 350 °C as thermal cleavage releases CO_2_. Once the carboxyl groups are removed, the remaining aromatic framework is destabilized, resulting in a 90% mass loss observed at 476 °C. The TGA profile for Ga-MIL-116 reveals a slight reduction in mass (~ 4.0%) at 200 °C that suggests liberation of bound water molecules^35^. Between, 250 °C and 550 °C the coordinating MOF linker thermally decomposes, leading to an overall 57% mass loss. This is a significantly higher thermal decomposition temperature compared to free mellitic acid, further supporting MOF coordination of the organic linker. The introduction of silver nanoparticles resulted in a moderate increase in the thermal stability of both the AgNPs (Supplementary Figure S3) and Ga-MIL-116 MOF (Fig. 2c), with the Ga-MIL-116@AgNP composite showing a mass loss onset point of 260 °C and total mass loss of 47% at > 500 °C. Taken together, this suggests that AgNPs anchored onto, and within, the porous MOF scaffold serve to enhance the thermal stability of the complex and hybridized silver nanoparticles.

Finally, infrared spectroscopy was used to interrogate the interactions between Ga³⁺ ions, organic linkers and AgNPs by analyzing the symmetric (ʋs) and asymmetric (ʋas) stretching vibrations of the different materials (Fig. 2d)^35^. Mellitic acid was first analyzed as a reference and exhibited broad ʋ(O–H) stretching vibrations of protonated carboxylic groups centered at 2862 cm⁻¹. Intense bands at 1697 cm⁻¹ and 1255 cm⁻¹ were assigned to C = O and C–O stretching of the carboxylic acid groups, respectively. The band at 863 cm⁻¹ corresponds to out-of-plane C–H bending of the aromatic ring, consistent with typical aromatic carboxylic acids^36^. Upon MOF formation, Ga-MIL-116 displayed a shift of the symmetric and asymmetric carboxylate stretching bands to 1453 cm⁻¹ (ʋs(COO⁻)) and 1639 cm⁻¹ (ʋas(COO⁻)), confirming deprotonation and coordination of the linker to Ga³⁺ metal centers. The disappearance of the 1697 cm⁻¹ C = O band further supports full carboxylate coordination and resonance delocalization within the framework^37^. As an additional control, the FTIR spectrum of free citrate-stabilized AgNPs (Supplementary Figure S4) was collected and showed characteristic stretching vibrations of surface-bound carboxylate groups at 1604 cm⁻¹ and 1383 cm⁻¹, respectively, from the citrate stabilizing reagent. A broad band near 3450 cm⁻¹ is attributed to O–H stretching from surface hydroxyl groups and adsorbed water molecules. After AgNP incorporation into Ga-MIL-116, the carboxylate stretching bands shifted to lower wavenumbers (1440 and 1590 cm⁻¹). This red-shift indicates electronic redistribution and multivalent coordination interactions involving both Ga³⁺ nodes and surface silver atoms. The decrease in Δν (ʋas–ʋs) compared to pristine Ga-MIL-116 suggests modified coordination symmetry, likely due to interfacial interactions between the MOF carboxylate groups and AgNP surfaces. Additional bands observed in the 1000–1200 cm⁻¹ region are attributed to C–O stretching vibrations, while features below 900 cm⁻¹ arise from aromatic ring deformation modes from the linker. The broad absorption centered at 3521 cm⁻¹ in both Ga-MIL-116 and Ga-MIL-116@AgNP is assigned to coordinated or hydrogen-bonded water molecules within the framework.

**Fig. 3:**
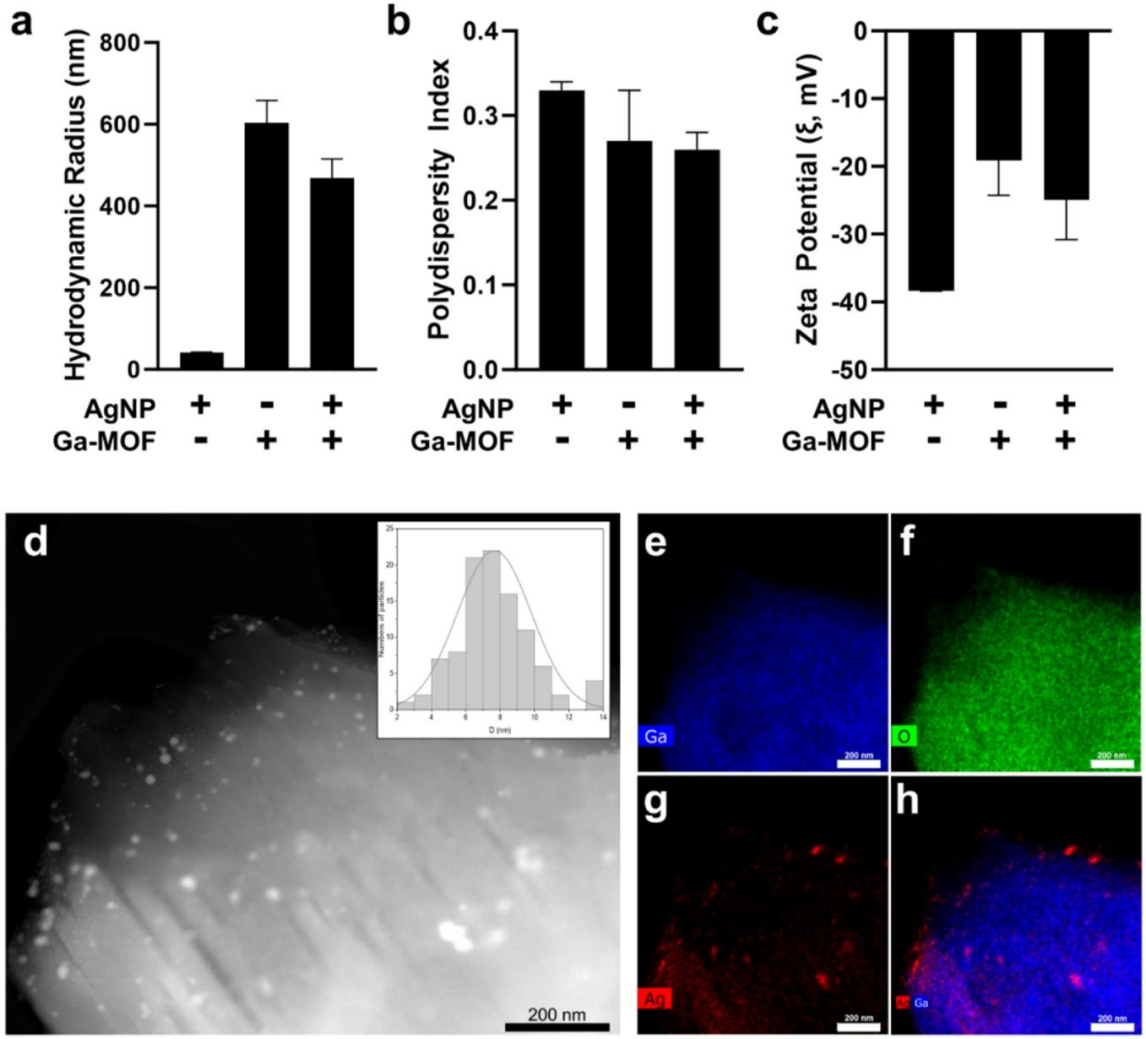
(**a**–**c**) Hydrodynamic radius (**a**), polydispersity (**b**) and zeta potential surface charge (**c**) of AgNPs, Ga-MIL-116 (Ga-MOF), or the hybrid material (Ga-MIL-116@AgNPs). (**d**–**h**) Transmission Electron Microscopy-Energy Dispersive X-ray Spectroscopy (TEM-EDS) imaging of Ga-MIL-116@AgNPs. (**a**) High-angle Annular Dark-field image. Scale bar = 200 nm. *Inset*: Frequency histogram of AgNP cluster diameter adsorbed to the surface of Ga-MIL-116 MOFs. Gallium (**e**), oxygen (**f**) and silver (**g**) elemental mapping, as well as the merge of the gallium and silver elemental signals (**h**).

Next, dynamic light scattering (DLS) studies were performed to evaluate the hydrodynamic size and surface charge of the synthesized materials. Results show pristine Ga-MIL-116 exhibited a hydrodynamic diameter of 603.69 ± 54.82 nm with a polydispersity index (PDI) of 0.27 ± 0.06, indicating a reasonably homogeneous size distribution (Fig. 3a, b). Upon incorporation of silver nanoparticles, the Ga-MIL-116@AgNPs composite displayed a reduced average hydrodynamic diameter of 468.38 ± 46.77 nm and a similar PDI (0.26 ± 0.02), suggesting that surface nucleation of AgNPs may displace mellitic acid ligands to generate smaller MOF frameworks. In contrast, free AgNPs presented a markedly smaller hydrodynamic size (41.96 ± 0.47 nm) and higher dispersity (PDI = 0.33 ± 0.01), consistent with the behavior of unconfined nanoparticles in suspension. Zeta potential measurements revealed that Ga-MIL-116 and Ga-MIL-116@AgNPs display negative surface charges of − 19.09 ± 5.20 mV and − 24.94 ± 5.89 mV, respectively (Fig. 3c). AgNPs alone exhibit the highest anionic surface charge (− 38.36 ± 0.05 mV). Given that particles with zeta potentials that exceed ± 20 mV are generally considered colloidally stable, these findings suggest that silver incorporation into Ga-MIL-116 MOFs not only reduces particle size but enhances the electrostatic stabilization of the hybrid MOF to improve their dispersion in aqueous physiological solutions.

Transmission electron microscopy coupled with energy-dispersive X-ray spectroscopy (TEM-EDS) was next employed to investigate the morphology and elemental distribution of Ga-MIL-116@AgNPs. The HAADF-STEM micrograph shown in Fig. 3d highlights the crystalline framework of the Ga-MIL-116 MOF decorated with silver nanoparticles. Analysis of surface-anchored AgNP size shows an average particle diameter of approximately 7.5 nm, with a narrow size distribution (Supplementary Figure S5). Elemental mapping of TEM results further confirmed homogeneous distribution of Ga throughout the MOF matrix (Fig. 3e), with co-localization of the metal signal and oxygen (O) from the coordinated mellitic acid linker (Fig. 3f). Further mapping of Ag signals (Fig. 3g), together with its overlap of Ga distribution (Fig. 3h), indicates that the silver nanoparticles are predominantly anchored to the external surface of the MOF crystallites, with potential shallow penetration into near-surface pores. Additional energy-dispersive X-ray spectrometry analysis further confirms the complex arrangement of Ga, O, and Ag elements within the hybrid Ga-MIL-116@AgNPs composite (Supplementary Figure S6). Taken together, these results suggest a post-synthetic deposition mechanism onto the MOF surface, rather than lattice incorporation, consistent with the absence of intense Ag reflections in the XRD data. Surface-localized AgNPs are particularly advantageous for antimicrobial activity, as they ensure interfacial accessibility and efficient contact with biologic targets. Additional stability assays in artificial saliva confirmed that silver nanoparticles remain bound to the MOF surface in oral environments (≤ 7% Ag^+^ release over 24 h, Supplementary Figure S7), ensuring MOFs display a high local concentration of antimicrobial silver during bacterial engagement in the oral cavity.

**Fig. 4:**
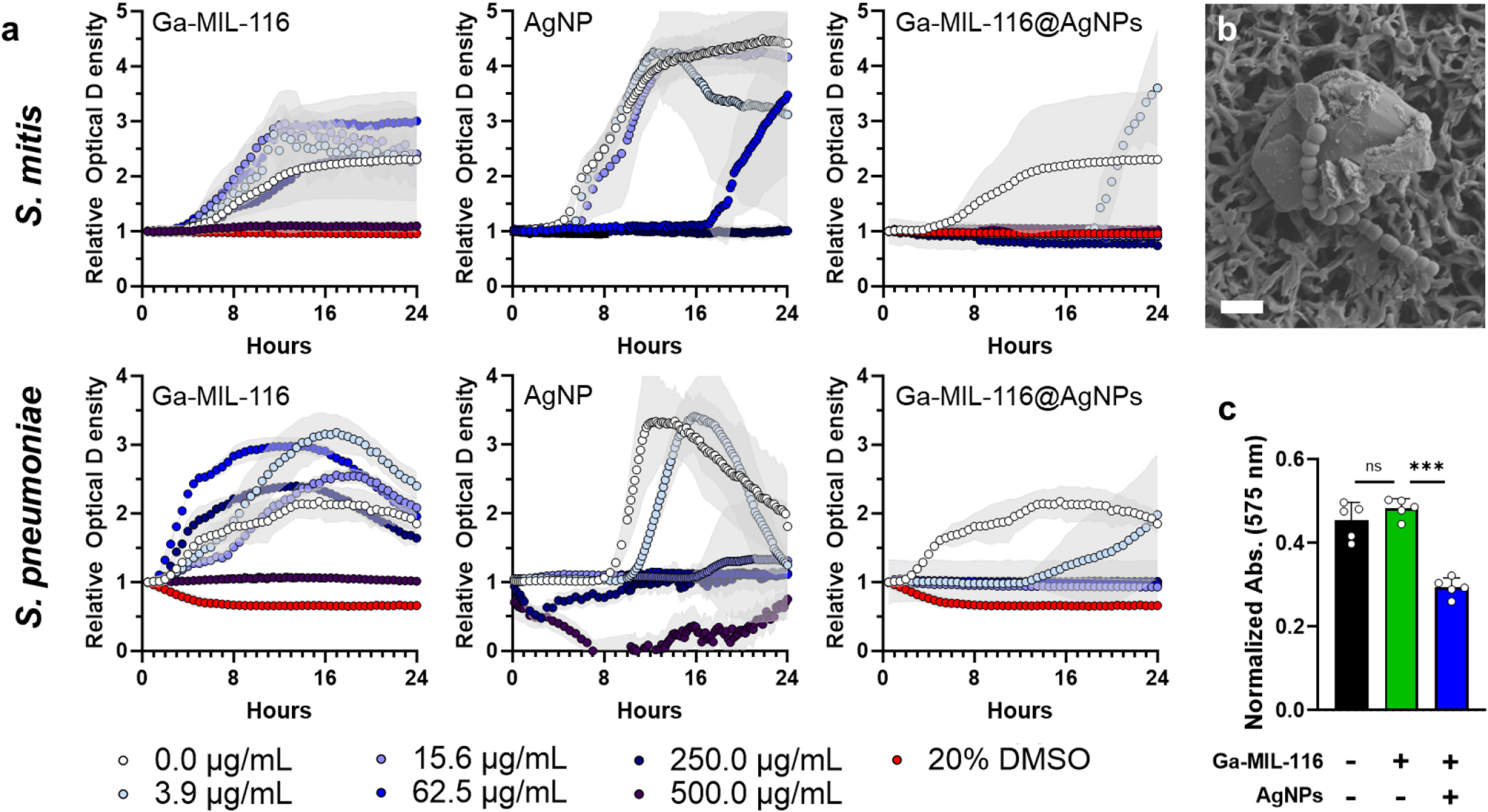
(**a**) Time-dependent optical density of *S. mitis* (top) and *S. pneumoniae* (bottom) microbes grown in the presence of Ga-MIL-116 (left), free AgNPs (middle), or Ga-MIL-116@AgNPs (right). Untreated (0.0 µg/mL) and 20% DMSO treated samples were included as negative and positive controls, respectively. Results are normalized to sterile treatment controls to remove the contribution of material optical density, with bacterial growth subsequently reported as relative optical density. Standard deviations of the curves are shown by the respective light gray colored overlay. The complete concentration series can be found in Supplementary Figure S8. (**b**) Scanning electron micrograph of *S. pneumoniae* interactions with a Ga-MIL-116@AgNP crystalline MOF. Scale bar = 2 μm. (**c**) Residual *S. mitis* biofilm biomass on hydroxyapatite enamel discs following a 24 h treatment with blank broth (-/-), Ga-MIL-116 (+/-) or Ga-MIL-116@AgNPs (+/+) at an equivalent particle concentration of 62.5 µg/mL, as determined via the crystal violet assay. Statistical significance determined via one-way ANOVA, with ns = not significant, *** *p* < 0.001.

The antibacterial synergy of Ga^3+^ and Ag^+^ ions was next tested in the model oral pathogens *S. mitis* and *S. pneumoniae* following treatment with silver free Ga-MIL-116 and silver containing Ga-MIL-116@AgNPs MOFs at 0–500 µg/mL concentrations (Fig. 4a). These pathogens were selected based on their role in pathologic biofilm formation on tooth pellicles, and potential to cause severe oral abscesses and maxillofacial complications^38–40^. Time-dependent optical density based growth studies demonstrate that the native Ga-MOF scaffold (Ga-MIL-116) is weakly active towards both microbes, with growth inhibition only observed at the highest tested concentration (500 µg/mL). In fact, concentrations of Ga-MIL-116 below 250 µg/mL appeared to marginally promote bacterial growth over the untreated control (0 µg/mL). This stands in contrast to other MOFs that exert bactericidal effects via mechanical interactions,^41^. and suggests that either the stability of the Ga-MOF structure prevents significant release of inhibitory Ga^3+^ ions over the 24 h incubation period, or that released ions are not sufficiently potent to disrupt iron-dependent metabolism in the pathogen, or both. These observations indicate that an organic linker with lower Ga^3+^ binding affinity may create a less cohesive MOF framework that can rapidly release antibacterial ions; an assertion that will be explored in future studies. These results also rationalized the addition of antibacterial AgNPs to the MOF scaffold to enhance its activity.

Bacteriologic testing demonstrated that AgNPs alone show moderate antibacterial activity, with concentrations ≥ 125 µg/mL and ≥ 31.3 µg/mL yielding complete growth inhibition for *S. mitis* and *S. pneumonia*, respectively (Fig. 4a). Gratifyingly, combination of AgNPs with Ga-MIL-116 led to strong antibacterial efficacy, with 3.9 µg/mL concentrations delaying bacterial outgrowth of both test microbes by ≥ 10 h and 7.8 µg/mL causing complete growth inhibition (full concentration series can be found in Supplementary Figure S8). Further, we found that Ga-MIL-116@AgNPs maintain their antimicrobial efficacy in simulated saliva environments (Supplementary Figure S9). Taken together, these findings highlight the potential of Ga-MIL-116@AgNPs to rapidly clear bacterial pathogens in the oral cavity, and suggests that release of Ga^3+^ ions from Ga-MIL-116@AgNPs particles may synergistically enhance the activity of surface-bound silver relative to free AgNPs.

In addition to growth inhibition assays, SEM imaging was performed to visualize the interaction between *S. pneumoniae* and the Ga-MIL-116@AgNPs surface (Fig. 4b). The micrograph shows bacterial cells in direct physical contact with the crystalline MOF particles, supporting a contact-dependent antimicrobial mechanism. Notably, the bacteria appear adhered to regions enriched with AgNPs, consistent with enhanced membrane interaction and local ion release at the material-microbe interface. Although no significant membrane rupture is observable at this magnification, the close association suggests that the Ga-MIL-116@AgNPs act through a combined mechanism involving contact-mediated bacterial binding and localized inactivation. To further support this assertion, we performed ROS generation assays and live/dead staining experiments following bacterial treatment with Ga-MIL-116@AgNPs (Supplementary Figures S10-11). Results show that Ga-MIL-116@AgNPs do not appreciably generate active ROS over time periods commensurate with their antibacterial activity (Supplementary Figure S10), and that microbial engagement with the particle surface leads to bactericidal cell death through mechanisms involving membrane destabilization (Supplementary Figure S11). Taken in context with our growth inhibition data, these observations suggest that Ga-MIL-116@AgNPs elicit contact-dependent mechanisms of bactericidal activity, and that spatial clustering of AgNPs on the MOF surface increases the probability of nanoparticle-bacteria interactions to enhance antimicrobial efficacy relative to free AgNPs. Finally, to evaluate Ga-MIL-116@AgNP efficacy towards biofilms, *S. mitis* was cultured on the surface of tooth enamel-mimetic hydroxyapatite discs to form biofilm matrices before MOF treatment. Results shown in Fig. 4c demonstrate that a 24-hour exposure to Ga-MIL-116@AgNPs at 62.5 µg/mL leads to ~ 50% removal of established *S. mitis* biofilms, while the native Ga-MIL-116 scaffold is inactive.

**Fig. 5:**
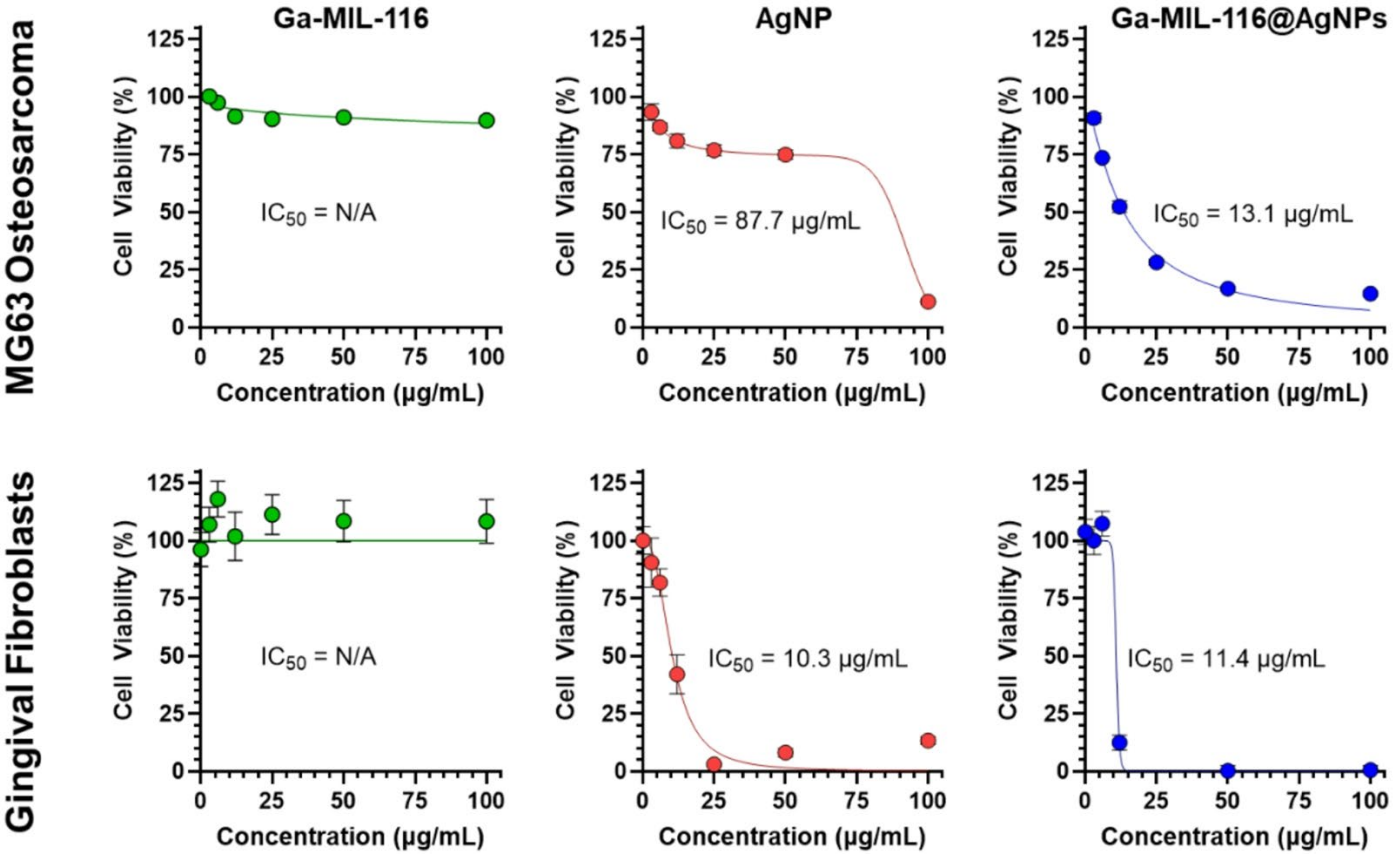
Viability of MG-63 human osteosarcoma and primary gingival fibroblasts cells treated with varying concentrations of Ga-MIL-116 (green), silver nanoparticles (AgNP, red) or Ga-MIL-116@AgNPs (blue). IC_50_ values are determined from a nonlinear regression fit of the data and reported in the appropriate panel.

Next, we investigated the cytotoxicity of the materials against both MG-63 human osteogenic and gingival fibroblast cells using MTT colorimetric metabolic assays as a surrogate for cell viability. Ga-MIL-116 was found to be generally well tolerated, with > 85% metabolic viability maintained after a 24-hour incubation at concentrations up to 100 µg/mL (Fig. 5). Conversely, free AgNPs led to more significant off-target effects, with concentrations > 6 µg/mL for gingival fibroblasts and > 25 µg/mL for MG-63 cells reducing viability below 80%. Combining the two materials to produce Ga-MIL-116@AgNPs led to similar effects, with concentrations of 11–13 µg/mL reducing cell viability ~ 50%. Given that Ga-MIL-116@AgNPs were effective against the streptococcal pathogens at concentrations at or above 3.9 µg/mL this suggests there is a narrow therapeutic window in which these compounds could be clinically effective as an oral therapeutic without harming host tissue. It is important to additionally mention that MG-63 is an osteosarcoma cell line, and Ga has well documented antineoplastic effects against malignant cells due to disrupting iron-dependent metabolic processes^42,43^. This may explain why Ga-MIL-116@AgNPs displayed enhanced toxicity towards MG-63 cancer cells relative to free AgNP controls.

## Conclusion

Metal nanoparticles continue to emerge as promising platforms for the development of topical oral anti-infective agents and antimicrobial dental materials. In this study, we demonstrate that hybrid gallium-silver MOFs rapidly engage with oral microbes and elicit potent bactericidal effects through contact dependent mechanisms of action. Notably, we found that Ga-MIL-116@AgNPs were able to completely inhibit the growth of *S. mitis* and *S. pneumoniae* at 7.8 µg/mL, outperforming both the native MOF (Ga-MIL-116) and free silver nanoparticles (AgNP). Cytocompatibility studies demonstrated that, although the parent Ga-MIL-116 MOF is generally well tolerated, the Ga-MIL-116@AgNP hybrid has dose limiting toxicity liabilities. This is a significant limitation of the current design and remains the focus of on-going work to improve its safety profile. Towards this goal, we plan to explore the incorporation of organic ligands with lower Ga interaction affinity, thereby facilitating the release of antibacterial Ga^3+^ ions that may synergistically enhance the activity of AgNPs. This, in turn, may improve antimicrobial potency and thereby enable lower dosing regimens to be employed that are better tolerated by host cells. Additional toxicity studies in primary oral keratinocytes and odontoblasts will provide further analysis of material safety. In sum, our findings suggest that, with further development, gallium-silver MOF hybrids may represent a promising topical antimicrobial platform for the localized treatment of odontogenic infections, and advocate for further preclinical material optimization and biologic evaluation.

## Supplementary Information

Supplementary Material 1.

## Data Availability

The data that support the findings of this study are available from the corresponding author upon reasonable request.
