# Supplementary material for: Antibacterial silver nanoparticle decorated gallium metal-organic frameworks for odontogenic infections
Source: Sci Rep. 2026 Apr 4;16:16235. doi: 10.1038/s41598-026-47319-7 (PMC13201781; doi:10.1038/s41598-026-47319-7)
Supplement: Supplementary file 1 — Supplementary Material 1. [file 41598_2026_47319_MOESM1_ESM.docx]

**Supporting Information**

**Antibacterial Silver Nanoparticle Decorated Gallium Metal-Organic Frameworks for Odontogenic Infections**

Fellype Diorgennes Cordeiro Gomes, Diptomit Biswas, Logan C. Eisaman, Mary Cristina Ferreira Alves, Severino Alves Júnior, Scott H. Medina

Content Page Number

**Supplementary Figure S1**. Diffractogram of AgNPs S2

**Supplementary** **Table S1.** Theoretical and experimental parameters of MOFs S3

**Supplementary Figure S2**. XPS spectra of Ga-MIL-116@AgNPs S4

**Supplementary Figure S3**. Thermogravimetric curve of Ag-NPs S5

**Supplementary Figure S4**. FTIR spectra of Ag-NPs S6

**Supplementary Figure S5**. Size histogram of MOF surface AgNPs S7

**Supplementary Figure S6**. MOF Energy-dispersive X-ray spectrum S8

**Supplementary Figure S7**. Silver release from Ga-MIL-116@AgNPs in artificial saliva S9

**Supplementary Figure S8**. Optical density bacterial growth curves S10

**Supplementary Figure S9**. Relative MIC of Ga-MIL-116@AgNPs in artificial saliva S11

**Supplementary Figure S10**. ROS generation of Ga MOFs in *S. mitis* S12

**Supplementary Figure S11**. Live/dead assay of *S. mitis* following Ga MOF treatment S13


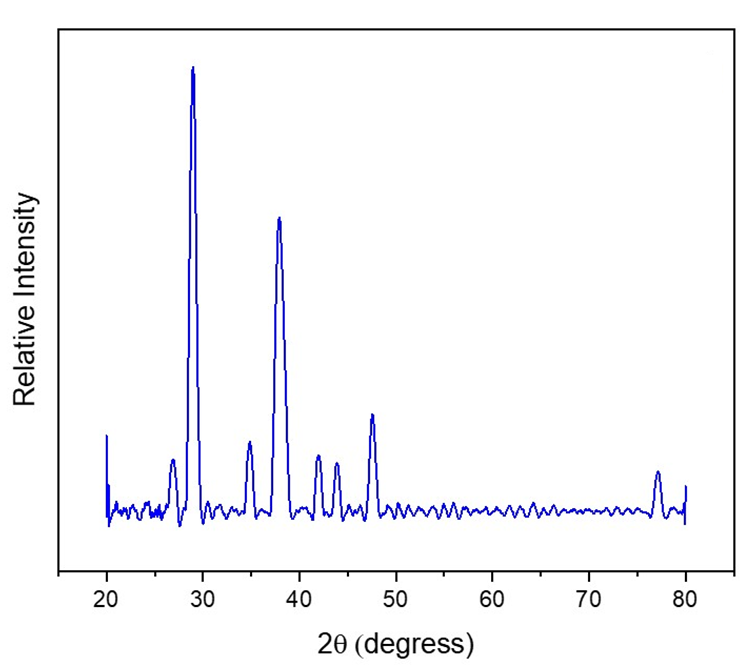


**Supplementary Fig. S1**. X-ray diffraction pattern of sodium citrate–stabilized silver nanoparticles (AgNPs). The diffraction peaks at 2θ ≈ 38.1°, 44.3°, 64.4°, and 77.4° are indexed to the (111), (200), (220), and (311) planes of face-centered cubic metallic silver (JCPDS No. 04-0783), confirming the crystalline nature of the AgNPs.

**Table S1.** Theoretical and experimental values ​​of lattice parameters and unit cell volumes of MOFs and AgNPs

| MOFs | Space Group | Crystalline System | Unit Cell Parameters | | | Cell Volume |
| --- | --- | --- | --- | --- | --- | --- |
| Ga-MIL-116*  (Theoretical) | *Cmcm* | orthorhombic | **a** (Å) | **b** (Å) | **c** (Å) | **V**(Å) |
|  |  |  | 11.7767 | 6.8342 | 17.7959 | 1432.2909 |
| Ga-MIL-116  (Experimental) | *Cmcm* | orthorhombic | 11.4998 | 6.7289 | 17.4898 | 1353.4006 |
| Ga-MIL-116@AgNPs (Experimental) | *Cmcm* | orthorhombic | 11.5743 | 6.7030 | 17.6594 | 1370.0788 |
| AgNPs  (Experimental) | *Fm-3m* | cubic | 4.09882 | 4.09882 | 4.09882 | 68.8613 |

*CCDC-929715


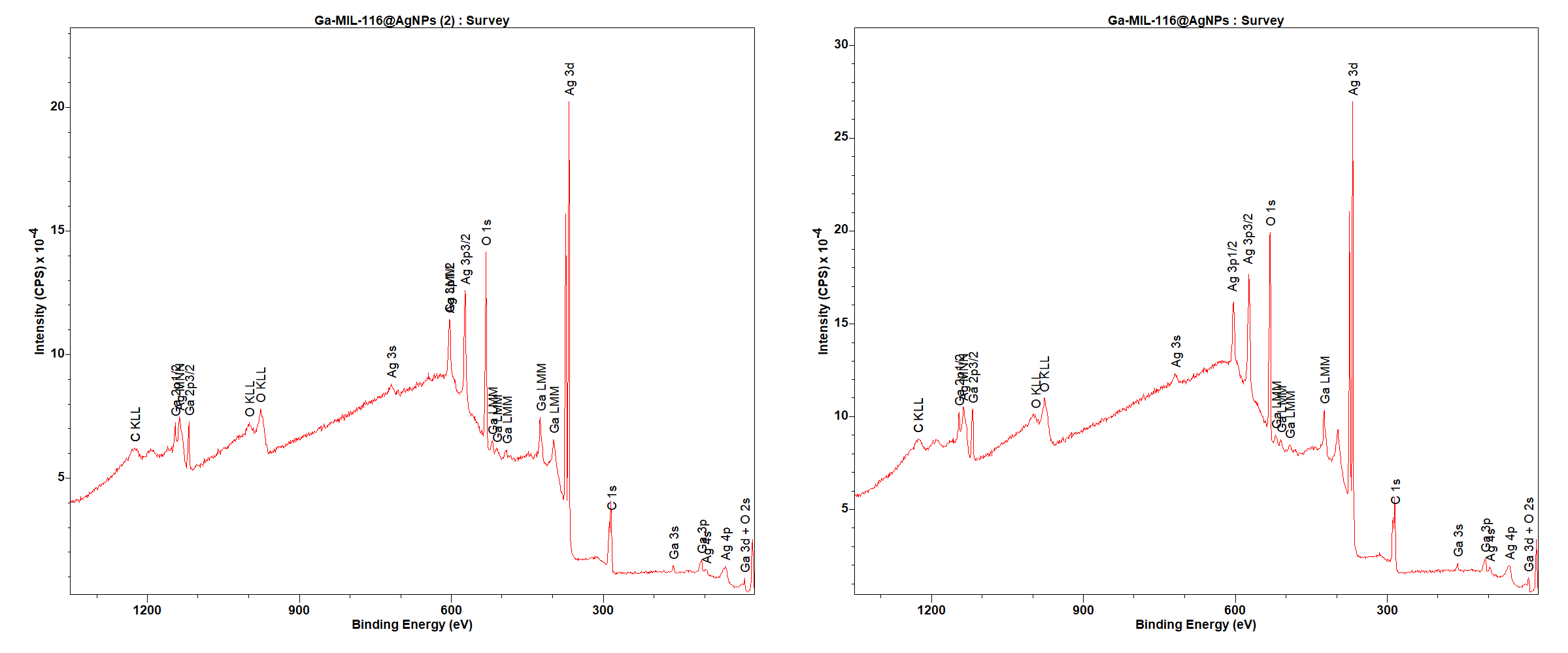


|  |  | |  |  | |  | |  | *%C species of Total C from fits of C 1s spectra.* | | | | |
| --- | --- | --- | --- | --- | --- | --- | --- | --- | --- | --- | --- | --- | --- |
| **Sample** | **Ag** | **Ga** | | | **O** | | **Total C** | | **Carbide** | **CHx-CHx** | **C-O** | **COO** | **CO_3_** |
| Ga-Mil-116 | N/D | 7.0 | | | 50.4 | | 42.6 | | 2.8 | 20.5 | 5.6 | 10.4 | 6.1 |
| Ga-MIL-116@AgNPs | 12.7 | 3.2 | | | 40.6 | | 43.5 | | N/D | 20.9 | 5.7 | 10.6 | 6.2 |

**Supplementary Fig. S2**. XPS spectra and calculated relative surface composition of the indicated elements for Ga-MIL-116@AgNPs. N/D = not detectable.

**
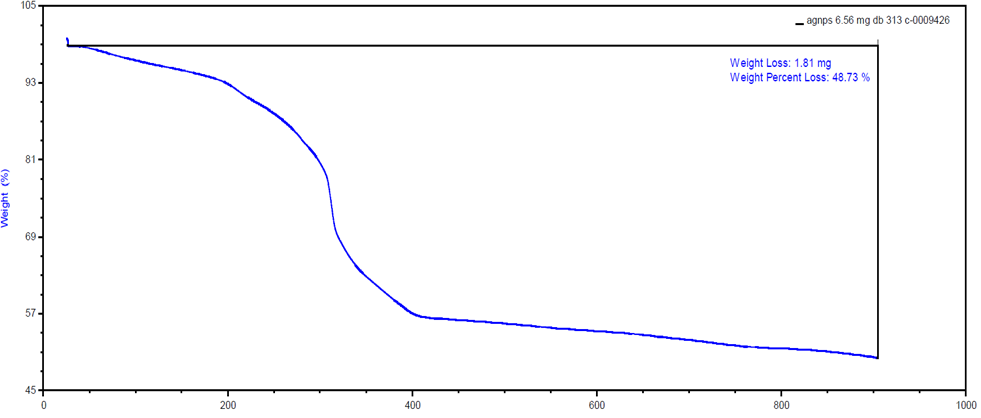
**

**Supplementary Fig. S3**. Thermogravimetric curve of free AgNPs.

**
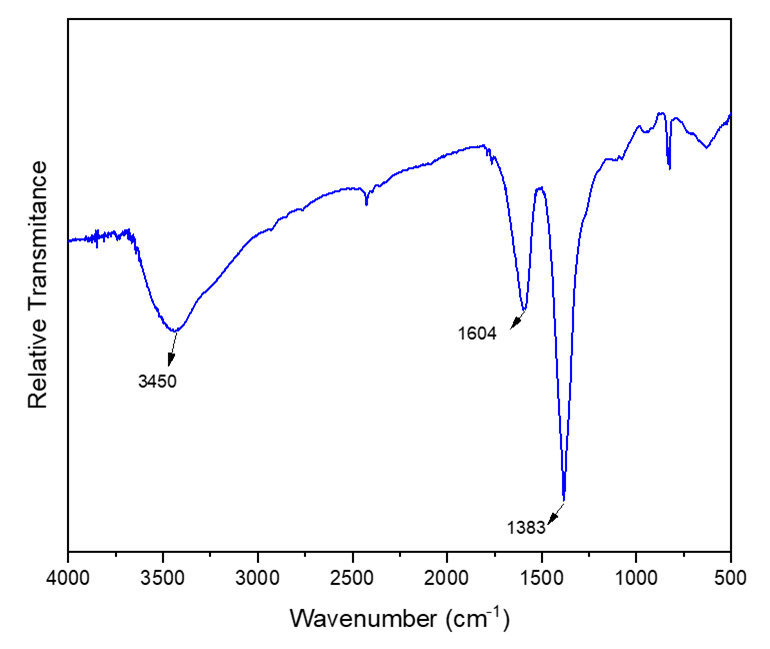
**

**Supplementary Fig. S4**. Fourier transform infrared adsorption spectra of sodium citrate–stabilized silver nanoparticles (AgNPs).

**Supplementary Fig. S5.** Histogram of silver nanoparticle diameters on the Ga-MIL-116 MOF surface (n = 100).


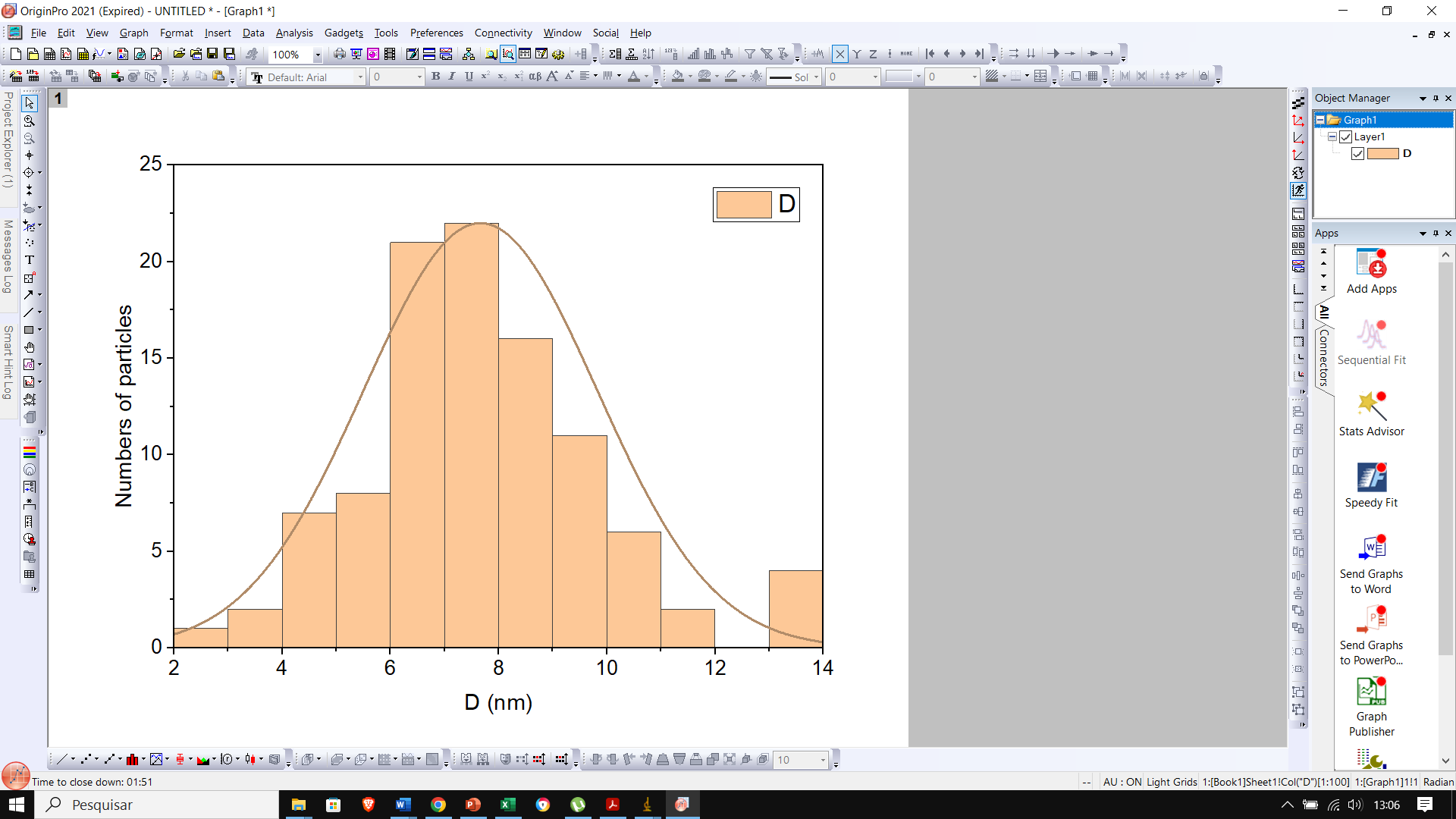


**Supplementary Fig. S6.** Energy-dispersive X-ray spectrum confirming Ga, O and Ag in Ga-MIL-116@AgNPs. Cu signals arise from the TEM support grid.


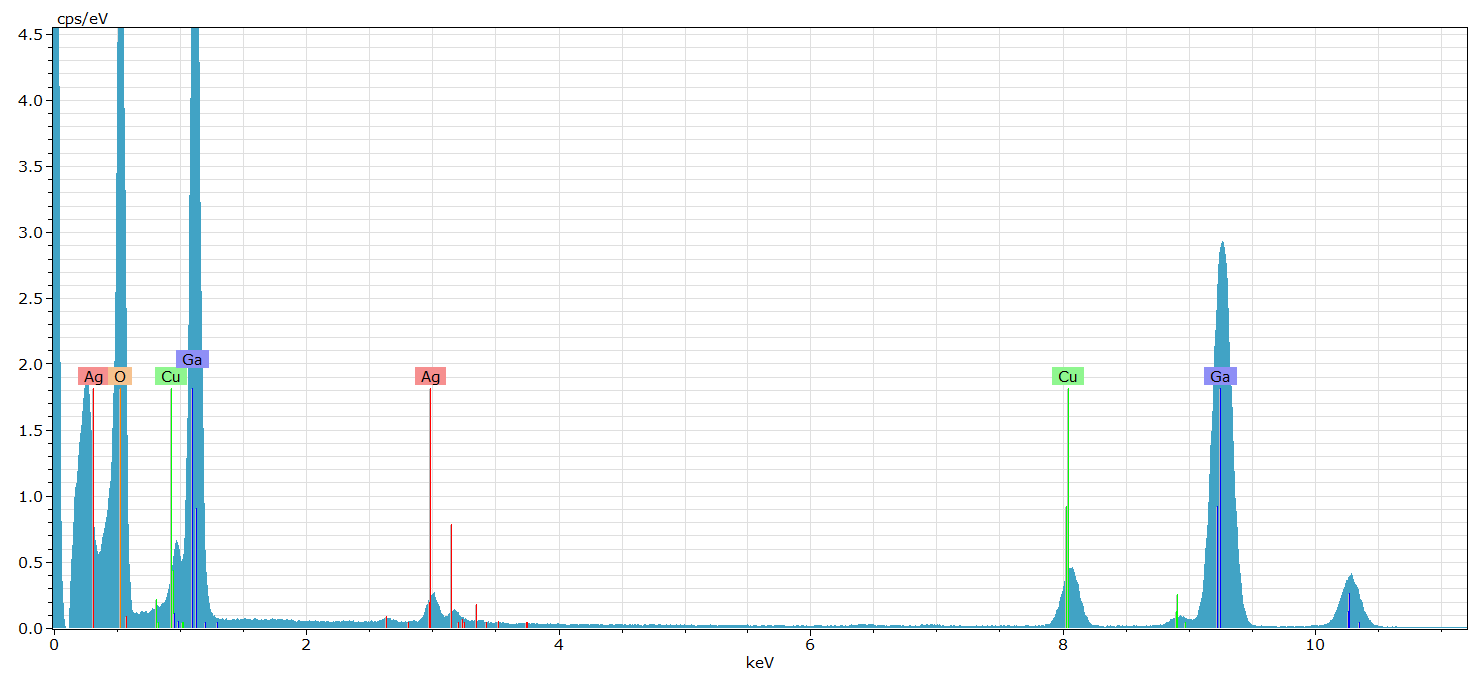


**Supplementary Fig. S7.** Release of Ag^+^ ions from the Ga-MIL-116@AgNP surface following a 2 – 24 hour incubation in water or artificial saliva (AS) at pH 5.5 or 7.0, without/with the hydrolytic saliva enzyme α-amylase (20 U/mL). The maximum equivalent mass of free silver from Ga-MIL-116@AgNPs was used as a positive control (62.5 µg).

**
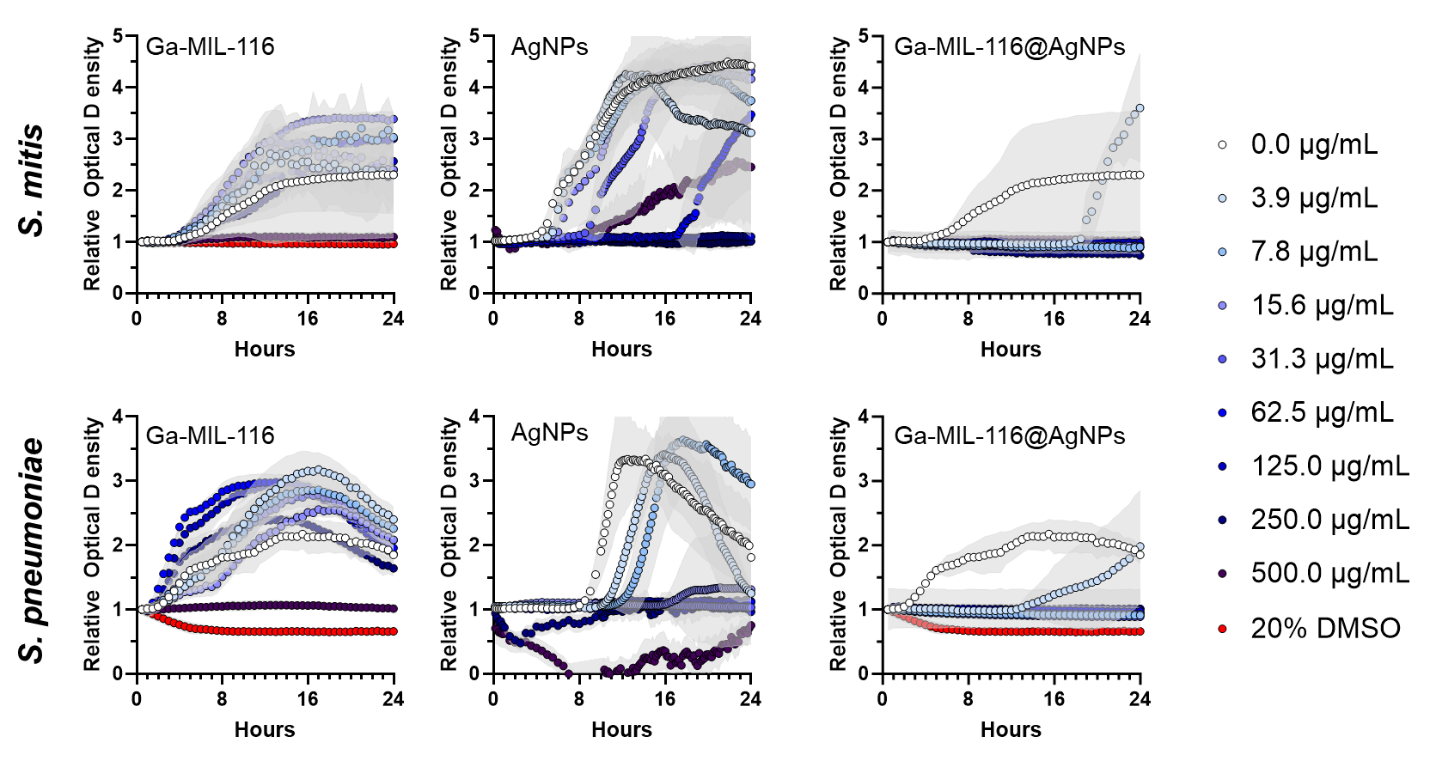
Supplementary Fig. S8:** Time-dependent relative optical density of *S. mitis* (top) and *S. pneumoniae* (bottom) microbes grown in the presence of Ga-MIL-116 (left), free AgNPs (middle), or Ga-MIL-116@AgNPs (right). Untreated (0.0 μg/mL) and 20% DMSO treated samples included as negative and positive controls, respectively. Standard deviations of the curves are shown by the respective light gray colored overlay.

**Supplementary Fig. S9.** Relative MIC of Ga-MIL-116@AgNPs towards *S. mitis* following a 2 – 24 hour pretreatment of the particles in water or artificial saliva (AS) at pH 5.5 or 7.0, without/with the hydrolytic saliva enzyme α-amylase (20 U/mL). Statistical significance determined via two-way ANOVA, with * p < 0.05.

**
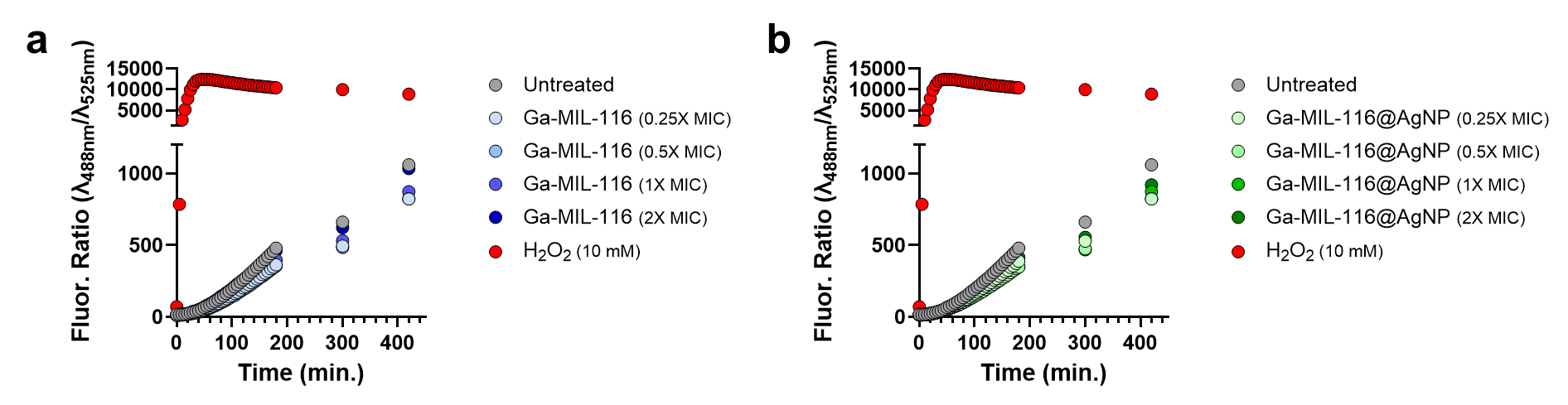
Supplementary Fig. S10:** Time-dependent generation of reactive oxygen species (ROS) within *S. mitis* following addition of Ga-MIL-116 (a, blue) or Ga-MIL-116@AgNPs (b, green) at varying MIC ratios. ROS generation was monitored using the cell permeable, oxidation sensitive dye DCFH-DA, as measured by the ratio of fluorescence at 488 nm and 525 nm (λ_488nm_/λ_525nm_). Blank solutions (untreated, gray) or 10mM H_2_O_2_ (red) were used as negative and positive controls, respectively.

**
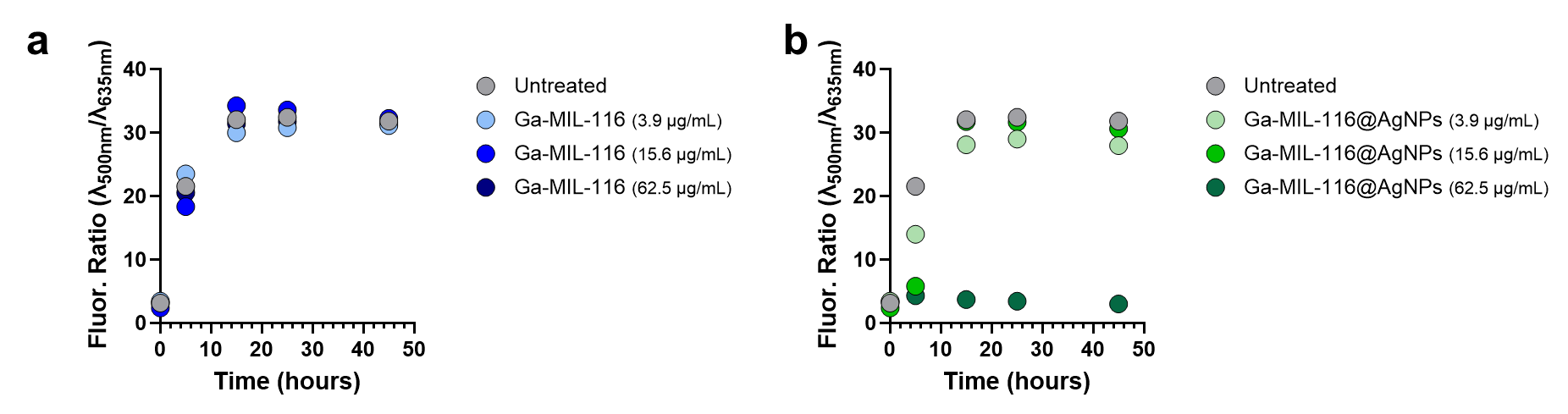
**

**Supplementary Fig. S11:** Time-dependent live/dead assay in *S. mitis* following addition of Ga-MIL-116 (a, blue) or Ga-MIL-116@AgNPs (b, green) at varying concentrations. Results are plotted as ratio of the live (SYTO9, λ_500nm_) and membrane-impermeable dead (propidium iodide, λ_635nm_) fluorophore reporter signal.
